# Early-to-Mid Gestation Fetal Testosterone Increases Right Hand 2D∶4D Finger Length Ratio in Polycystic Ovary Syndrome-Like Monkeys

**DOI:** 10.1371/journal.pone.0042372

**Published:** 2012-08-22

**Authors:** Andrew D. Abbott, Ricki J. Colman, Ross Tiefenthaler, Daniel A. Dumesic, David H. Abbott

**Affiliations:** 1 Wisconsin National Primate Research Center, University of Wisconsin, Madison, Wisconsin, United States of America; 2 Department of Obstetrics and Gynecology, University of Wisconsin, Madison, Wisconsin, United States of America; 3 Endocrinology-Reproductive Physiology Program, University of Wisconsin, Madison, Wisconsin, United States of America; 4 Department of Obstetrics and Gynecology, David Geffen School of Medicine at the University of California Los Angeles, Los Angeles, California, United States of America; University of Turku, Finland

## Abstract

A smaller length ratio for the second relative to the fourth finger (2D∶4D) is repeatedly associated with fetal male-typical testosterone (T) and is implicated as a biomarker for a variety of traits and susceptibility to a number of diseases, but no experimental human studies have been performed. The present study utilizes the rhesus monkey, a close relative of humans, and employs discrete gestational exposure of female monkeys to fetal male-typical T levels for 15–35 days during early-to-mid (40–76 days; n = 7) or late (94–139 days; n = 7) gestation (term: 165 days) by daily subcutaneous injection of their dams with 10 mg T propionate. Such gestational exposures are known to enhance male-typical behavior. In this study, compared to control females (n = 19), only early-to-mid gestation T exposure virilizes female external genitalia while increasing 2D∶4D ratio in the right hand (RH) by male-like elongation of RH2D. RH2D length and 2D∶4D positively correlate with androgen-dependent anogenital distance (AG), and RH2D and AG positively correlate with duration of early-to-mid gestation T exposure. Male monkeys (n = 9) exhibit a sexually dimorphic 2D∶4D in the right foot, but this trait is not emulated by early-to-mid or late gestation T exposed females. X-ray determined phalanx measurements indicate elongated finger and toe phalanx length in males, but no other phalanx-related differences. Discrete T exposure during early-to-mid gestation in female rhesus monkeys thus appears to increase RH2D∶4D through right-side biased, non-skeletal tissue growth. As variation in timing and duration of gestational T exposure alter male-like dimensions of RH2D independently of RH4D, postnatal RH2D∶4D provides a complex biomarker for fetal T exposure.

## Introduction

The sexually dimorphic digit ratio between the length of the second (index) finger and the length of the fourth (ring) finger (2D∶4D) has long been established as smaller in men compared to women [1]. Fetal testosterone (T) exposure spanning early-to-mid gestation [2] has been repeatedly implicated in the development of sexual dimorphism in 2D∶4D [3]–[6]. This finger length ratio has achieved prominence because of its association with a variety of human diseases in men and women: men, prostate, testicular and oral cancer [7]–[9], infertility [8], autism [10], attention deficit disorder [11] and eating disorders [12]; women, breast and cervical cancer [13], [14], autism [15], [16], congenital adrenal hyperplasia (CAH) [5], and polycystic ovary syndrome (PCOS) [17]. Due to the apparent influence of fetal T exposure on 2D∶4D and the latter's association with human disease, 2D∶4D has been proposed as a faithful postnatal biomarker for gestational exposure to T and its associated risk of pathophysiology [3], [18], [19].

Supporting evidence for fetal testosterone differentiation of 2D∶4D in humans is provided by genetically-determined androgenic abnormalities, including classical CAH – a hypocortisolemic condition commonly caused by 21-hydroxylase deficiency, that exposes fetuses to abnormally high levels of adrenal androgens [20], [21]. Women with classic (early gestation onset [22]) CAH exhibit masculinized physical and behavioral characteristics as well as lower, more male-like 2D∶4D [5]. Buck and colleagues [23], however, using only left hand measures that show a less pronounced differential in 2D∶4D [3], [24], [25], fail to show a smaller 2D∶4D in women with CAH. In contrast to 46,XX CAH individuals, 46,XY individuals suffering from complete androgen insensitivity syndrome (CAIS) present with a female-like 2D∶4D [19] when T action is absent lifelong.

Thus, while associative findings from human studies are mostly supportive of the hypothesis that fetal male-typical T levels determine male-like 2D∶4D, a controlled, experimental study confirming fetal T action on 2D∶4D has not been performed. In this regard, the experimentally-controlled exposure of female rhesus monkeys (*Macaca mulatta*) to fetal male levels of T provides a nonhuman primate model in which to determine the 2D∶4D consequence of fetal exposure to a known duration of fetal male-typical T exposure [26]. Such T-exposed, prenatally androgenized (PA) female monkeys display more male-typical behavior [27]–[29], regardless of whether female fetuses are exposed to T during either early-to-mid or late gestation [28], [30], [31]. PA monkeys also show varying degrees of masculinized genitalia, including a male-like anogenital distance [30], [32], but only when fetal exposure to T is initiated during early gestation [28], [29], [33].

PA female rhesus monkeys exposed to T during either early-to-mid or late gestation also demonstrate signs and symptoms of PCOS, a common syndrome of T excess in women [34]–[37]. PCOS-like traits are most prominent in female monkeys exposed to fetal male-typical T levels during early-to-mid gestation and include ovarian and adrenal androgen excess, intermittent or absent menstrual cycles, polycystic ovaries, increased adiposity, hyperlipidemia, insulin resistance and impaired insulin secretion, as well as increased incidence of type 2 diabetes mellitus [34]–[40]. Such comprehensive reproductive, endocrine and metabolic sequelae of female fetal T exposure suggest pathophysiological developmental impact on multiple organ systems, potentially by means of epigenetic programming [41].

The objective of this pilot study is to determine whether fetal male-typical T exposure induces a smaller 2D∶4D ratio in a female monkey model for PCOS. Identifying fetal origins for such a lower ratio in a nonhuman primate could re-affirm 2D∶4D as a biomarker for both fetal T exposure and PCOS. Rhesus monkeys have a mildly smaller, sexually dimorphic 2D∶4D finger length ratio [24], and we hypothesize that developing females exposed *in-utero* to male-typical T at the end of the 1^st^ to mid-2^nd^ trimester (early-to-mid gestation) demonstrate a more masculinized 2D∶4D [2].

## Results

### Selected somatic, reproductive and endocrine characteristics of monkey groups

Aspects of adult phenotypic features related to fetal T exposure are summarized in Table 1. While none of the female groups differed with respect to age, body weight and BMI, males were slightly older than control (p<0.016, partial eta squared (*η* ^2^_p_) = 0.29; effect size [42]–[44]) and LPA (p<0.006, *η* ^2^_p_ = 0.29) females. Compared to all three female groups, however, and typical of rhesus monkey sexual dimorphism, males were heavier (p<2.6×10^−4^, *η* ^2^_p_ = 0.53), exhibited greater crown-rump length (p<0.033, *η* ^2^_p_ = 0.35), longer anogenital distance (p<1.8×10^−4^, *η* ^2^_p_ = 0.95), and had greater BMI (p<0.05, *η* ^2^_p_ = 0.26). Anogenital distance, a measure of early gestation T exposure, indicated that EPA females exhibited more male-typical dimensions than control or LPA females. EPA females, nevertheless, did not display the same extension of anogenital distance as manifest by males (p<1.8×10^−4^).

**Table 1:** Somatometric and PCOS-like traits, right hand 2^nd^ digit length and 2D∶4D finger length ratio, in control (n = 19), early (EPA, n = 7) and late (LPA, n = 7) prenatally androgenized female and control male (n = 9) rhesus monkeys.

	Control female	EPA	LPA	Male
Age (Years)	20.3±0.6^a^	21.5±0.9	19.0±0.9^m^	23.4±0.8
Body Weight (kg)	8.8±0.4^b^	8.6±0.6^e^	8.8±0.6^n^	12.6±0.5
BMI (kg/m^2^)	38.4±1.6^a^	36.5±2.6^c^	37.6±2.6^l^	47.3±2.4
CR Length (cm)	48.0±0.5^b^	48.7±0.8^c^	48.3±0.8^l^	51.8±0.7
AG Distance (mm)	18.4±5.0^b^	94.6±6.3^e^^,^ h ^,^ j	15.0±7.0^n^	151.7±5.3
Basal T (ng/mL)	0.21±0.05	0.34±0.04	0.27±0.05	n.a.
Hyperandrogenic (≥0.32 ng/mL)	0%	57%	≥43%	n.a.
Polyfollicular Ovaries	n.a.	71%	≥29%	n.a.
Menstrual Cycle (Days)	28 (27, 34)	52 (35, 70)^g^	39 (31, 62)^o^	n.a.
Intermittent/Anovulatory (%)	≥10%	86%	≥57%	n.a.
RH 2D Length (cm)	3.215±0.046^b^	3.443±0.067^f^	3.278±0.067^l^	3.569±0.055
RH 2D∶4D	0.817±0.012	0.884±0.014^d^^,^ i ^,^ k	0.815±0.015	0.799±0.011

CR: Crown-Rump, AG: Anogenital, Data are shown as mean ± SEM or median (range).

a Control Female<Male, 0.01<p<0.05.

b Control Female<Male, p<0.001.

c EPA<Male, 0.01<p<0.05.

d EPA<Male, 0.001<p<0.01.

e EPA<Male, p<0.001.

f EPA>Control Female, 0.01<p<0.05.

g EPA>Control Female, 0.001<p<0.01.

h EPA>Control Female, p<0.001.

i EPA>LPA, 0.01<p<0.05.

j EPA>LPA, p<0.001.

k EPA>Male, p<0.001.

l LPA<Male, 0.01<p<0.05.

m LPA<Male, 0.001<p<0.01.

n LPA<Male, p<0.001.

o LPA>Control Female, 0.01<p<0.05.

Adult female monkey traits relevant to a PCOS-like condition include high basal T levels, intermittent or absent menstrual cycles and the presence of polyfollicular ovaries. In this study, basal T levels from adult females during the early follicular phase of the menstrual cycle or anovulatory period, while similar between groups (p<0.18), averaged ∼50% higher in EPA compared to control females (Table 1). Four of seven EPA and three of seven LPA female basal testosterone levels met the previously established criteria for rhesus monkey hyperandrogenism (>1 SD above normal control population mean [41]), demonstrating the presence of adult female hyperandrogenism in 50% of PA monkeys. Intermittent or absent menstrual cycles were found in both PA female groups as evidenced by increased intervals (Table 1) between ovulatory menstrual cycles. Polyfollicular ovaries, identified by trans-abdominal illumination of individual ovaries during the early follicular phase or anovulatory period, showed 71% and ≥29% incidence in EPA and LPA groups respectively (>10, ∼1–3 mm follicles in one or both ovaries). Criteria for defining polyfollicular ovarian morphology were based on those for women [45] as monkey ovarian assessments were made prior to Rotterdam criteria (≥12 follicles in any one ovary [46]).

### Lengths and ratios of digits

**Figure 1:**
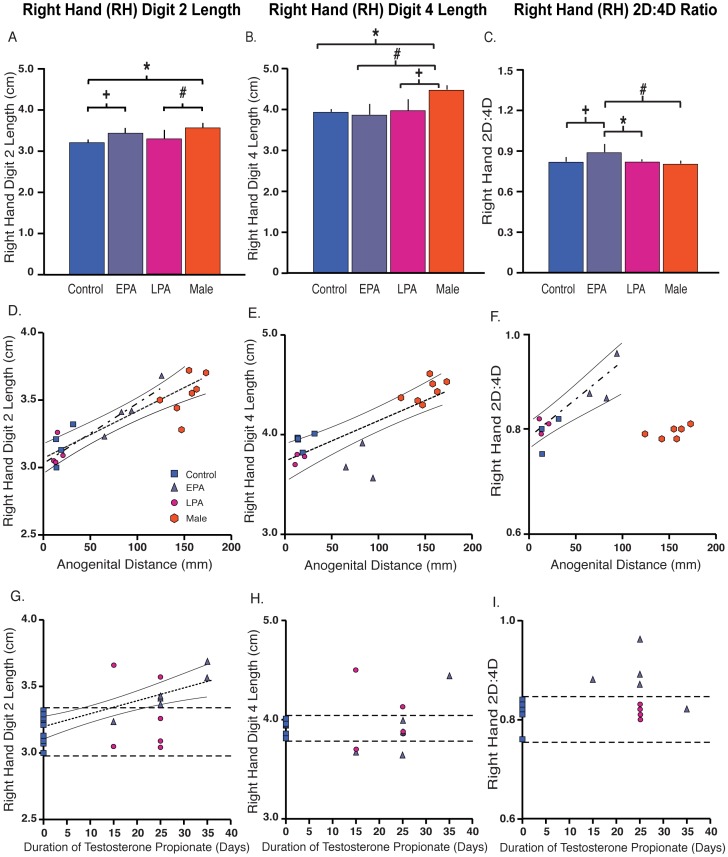
Right hand (RH) finger parameters in adult control and early (EPA) and late (LPA) prenatally androgenized female and male rhesus monkeys. (A) RH 2D finger length (^a^ p<2.9×10^−4^, Control<Male; ^b^ p<0.041, EPA>Control; ^c^ p<0.012, LPA<Male), (B) RH 4D finger length (^d^ p<3.6×10^−4^, Control<Male; ^e^ p<3.6×10^−4^, EPA<Male; ^f^ p<0.002; LPA<Male), (C) RH 2D∶4D ratio (^g^ p<6.6×10^−4^, EPA>Male; ^h^ p<0.009 EPA>Control Female; ^I^ p<0.016, EPA>LPA), (D) relationship between RH 2D finger length and anogenital distance (all groups, dashed line: r^2^ = 0.76, p<1.0×10^−6^; females only, dot-dash line: r^2^ = 0.79, p<1.0×10^−4^; 95% Confidence Interval (CI), solid lines), (E) relationship between RH 4D finger length and anogential distance (all groups, dashed line: r^2^ = 0.65, p<5.7×10^−5^; females only: n.s.; 95% CI, solid lines), (F) relationship between RH 2D∶4D ratio and anogenital distance (all groups: n.s.; females only, dot-dash line: r^2^ = 0.79, p<6.3×10^−4^; 95% CI, solid lines), (G) relationship between RH 2D finger length and duration of gestational exposure to testosterone propionate (Control and EPA females only, dotted line: r^2^ = 0.62, p<6.0×10^−5^; 95% CI, solid lines), (H) relationship between RH 4D finger length and duration of gestational exposure to testosterone propionate (Control and EPA females only: n.s.), and (I) the relationship between RH 2D∶4D ratio and duration of gestational exposure to testosterone propionate (Control and EPA females only: n.s.). Horizontal dashed lines indicate range of control female values (G–I).

**Figure 2:**
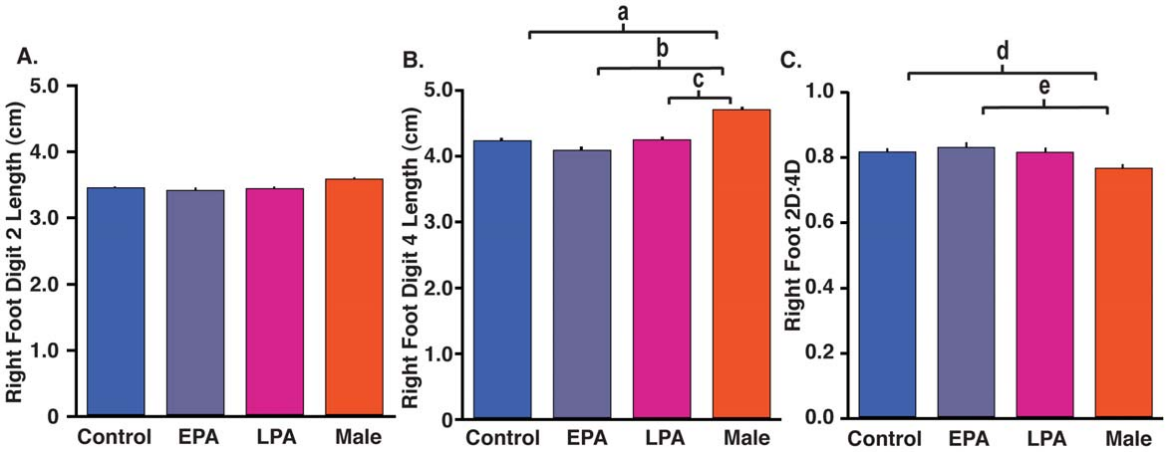
Right foot (RF) toe parameters in adult control and early (EPA) and late (LPA) prenatally androgenized female and male rhesus monkeys. (A) RF 2D finger length, (B) RF 4D finger length (^a^ p<9.0×10^−4^, Control Female<Male; ^b^ p<0.002, EPA<Male; ^c^ p<0.011, LPA<Male), and (C) RF2D∶4D ratio (^d^ p<0.02, Control Female>Male; ^e^ p<0.04, EPA>Male).

As typical for rhesus monkeys, all digit lengths in males were longer (p≤0.006) than those in control females, except for 2D on the right foot (Table 2). The length of right hand 2D in EPA females was male-like, exhibiting increased length compared to control females, and being comparable in length to males (Figure 1a). Five of seven EPA, but only two of seven LPA, females exceeded control values for right hand 2D length. As there was no extension of the right hand 4D length in PA females (Figure 1b), right hand 2D∶4D in EPA females exceeded that of control and LPA females, as well as that of males (Figure 1c). Remaining digit length ratios did not differ between male and female groups (Table 3, Figure 2), except for 2D∶4D and 2D∶3D in the right foot. In this latter regard, males showed an expected, sexually dimorphic smaller 2D∶4D ratio compared to control females (Table 3, Figure 2) and emulated that sex differential in 2D∶3D. Interestingly, EPA females showed no difference to controls.

**Table 2:** Mean (± SEM) digit length in control, early (EPA) and late (LPA) prenatally androgenized females and control male rhesus monkeys.

Digit length	Control female	EPA	LPA	Male
**Left hand**				
2D	3.219±0.056^a^	3.340±0.096	3.349±0.081	3.615±0.076
3D	4.090±0.076^b^	4.232±0.127^c^	4.134±0.114^i^	4.690±0.090
4D	3.969±0.065^b^	4.134±0.104	4.080±0.095^g^	4.480±0.082
**Right hand**				
2D	3.215±0.046^b^	3.443±0.067^f^	3.278±0.067^g^	3.569±0.055
3D	4.083±0.065^b^	4.115±0.097^c^	-*	4.543±0.074
4D	3.932±0.073^b^	3.858±0.092^e^	3.966±0.092^h^	4.464±0.073
**Left foot**				
2D	3.230±0.061^b^	3.428±0.086	3.522±0.106	3.669±0.075
3D	4.413±0.065^a^	4.500±0.112	4.387±0.102^g^	4.812±0.083
4D	4.168±0.071^a^	4.240±0.101^c^	4.265±0.109	4.614±0.089
**Right foot**				
2D	3.425±0.059	3.386±0.112	3.413±0.103	3.557±0.084
3D	4.420±0.066^b^	4.532±0.131	4.466±0.117	4.831±0.093
4D	4.206±0.065^b^	4.055±0.130^d^	4.215±0.106^g^	4.678±0.087

* There were only two LPA females with intact phalanges in the 3^rd^ digit of their right hand.

a Control Female<Male, 0.001<p<0.01.

b Control Female<Male, p<0.001.

c EPA<Male, 0.01<p<0.05.

d EPA<Male, 0.001<p<0.01.

e EPA<Male, p<0.001.

f EPA>Control Female, 0.01<p<0.05.

g LPA<Male, 0.01<p<0.05.

h LPA<Male, 0.001<p<0.01.

i LPA<Male, p<0.001.

**Table 3:** Mean (± SEM) digit length ratio in control, early (EPA) and late (LPA) prenatally androgenized female and control male rhesus monkeys.

Digit length ratio	Control female	EPA	LPA	Male
**Left hand**				
2D∶3D	0.794±0.010	0.800±0.019	0.806±0.015	0.769±0.012
2D∶4D	0.817±0.010	0.830±0.017	0.828±0.014	0.806±0.012
3D∶4D	1.032±0.014	1.017±0.017	1.000±0.015	1.049±0.010
**Right hand**				
2D∶3D	0.795±0.010	0.830±0.014	-*	0.784±0.011
2D∶4D	0.817±0.012	0.884±0.014^b^^,^ c ^,^ e	0.815±0.015	0.799±0.011
3D∶4D	1.042±0.014	1.060±0.017	-*	1.019±0.011
**Left foot**				
2D∶3D	0.747±0.013	0.776±0.018	-*	0.769±0.014
2D∶4D	0.790±0.012	0.802±0.017	-*	0.805±0.014
3D∶4D	1.053±0.010	1.038±0.016	1.030±0.014	1.044±0.012
**Right foot**				
2D∶3D	0.767±0.006^a^	0.778±0.012^d^	0.772±0.012^f^	0.735±0.008
2D∶4D	0.811±0.010^a^	0.825±0.019^d^	0.810±0.015	0.761±0.013
3D∶4D	1.059±0.008	1.090±0.017	1.050±0.013	1.036±0.011

* There were only two LPA females with intact phalanges permitting calculation of 2D∶3D and 3D∶4D in the right hand together with 2D∶3D and 2D∶4D in the left foot.

a Control Female>Male, 0.01<p<0.05.

b EPA>Control Female, 0.001<p<0.01.

c EPA>LPA, 0.01<p<0.05.

d EPA>Male, 0.01<p<0.05.

e EPA>Male, p<0.001.

f LPA>Male, 0.01<p<0.05.

### Lengths and ratios of phalanges and joint space width

Phalanx length and joint space width showed less pronounced sexual dimorphism than digit length (Table S1, data not shown, respectively). There was no sexual dimorphism in any phalanx length ratio (Table S2). There were no between female group differences in any phalanx or joint space width measurements.

### Associations with duration of fetal T exposure

**Figure 3:**
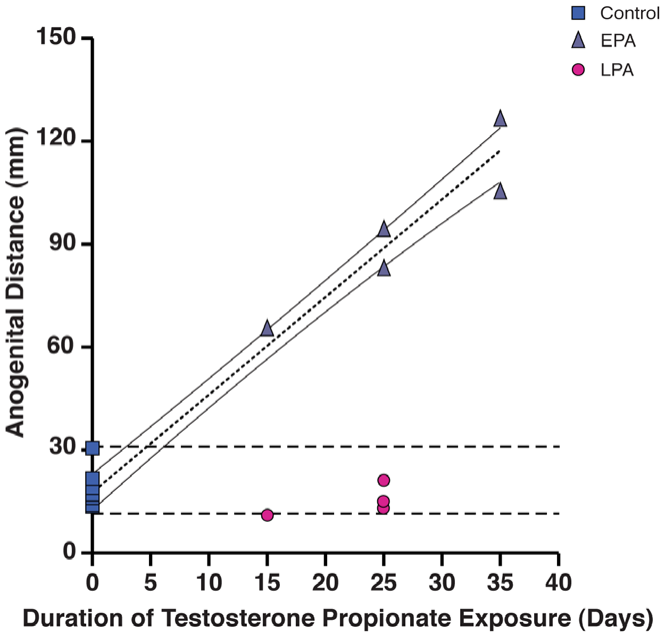
Female anogenital distance positively correlates (Control and early prenatally androgenized (EPA) females only, dotted line: r^2^ = 0.97, p<1.0×10^−6^; 95% CI, solid lines) with duration of early-to-mid gestation fetal T exposure. Horizontal dashed lines indicate range of control female values.

Both anogenital distance (Figure 3: r^2^ = 0.97, p<1.0×10^−5^) and right hand 2D length (Figure 1g: r^2^ = 0.62, p<6.0×10^−5^) were positively influenced by the duration of fetal T exposure in EPA, but not LPA, females. No associations were found with right hand 4D length (Figure 1h), right hand 2D∶4D (Figure 1i) or other finger, toe and phalanges, and joint space width parameters (data not shown).

### Associations between anogenital distance and right hand 2D and 2D∶4D

Figure 1d illustrates the positive association (r^2^ = 0.79, p<1.0×10^−4^ between all female groups; r^2^ = 0.76, p<1.0×10^−6^ between all groups) linking anogenital and right hand 2D elongation. EPA females, with only early-to-mid gestation exposure to fetal male levels of T, are found in an intermediate position between control and LPA females, and normal males, for both parameters (Figure 1d). There was no relationship (r^2^ = 0.22, p>0.14), however, between anogenital distance and right hand 4D length for female groups alone (Figure 1e). When including males with all female groups, right hand 4D length positively correlated with anogenital distance (Figure 1e: r^2^ = 0.65, p<5.7×10^−5^). A positive relationship (r^2^ = 0.79, p<6.3×10^−4^) between anogenital distance and right hand 2D∶4D is found when only female groups are considered (Figure 1f).

## Discussion

In many studies, a smaller 2D∶4D finger length ratio is repeatedly proposed as a reliable adult biomarker of fetal T exposure [3]–[5], [7], [47]–[51]. Developmental sex differences in 2D∶4D start as early as 14 weeks of gestation in humans, at the beginning of the second trimester [52], [53], [54], and do not stabilize until at least two years of age [3]. The bony phalanges within the second digit, however, can increase throughout childhood, though only in the left hand [2]. Fetal T may enable preferential digit growth by stimulating the expression of a series of digit development genes, *Wnt5*, *Ihh*, *Bmp6*, *Fgrf2*, *Igfbp2/5*, *Sox9* and *Runx2* [25], or through interaction with sexually dimorphic, differential expression of androgen and estrogen receptors [25].

### Fetal T exposure induces changes in 2D∶4D in female rhesus monkeys

The current pilot study is the first to experimentally manipulate a nonhuman primate to test the hypothesis that fetal T exposure differentiates a smaller 2D∶4D. Fetal female rhesus monkeys exposed to fetal male levels of T during either early-to-mid gestation (EPA monkeys) or mid-to-late gestation (LPA monkeys) [55], [56] exhibit a variety of masculinized behavioral, physical and physiological traits including, respectively, mounting behavior, virilized genitalia and impaired steroid negative feedback on luteinizing hormone [28]–[32], [36]. As anticipated from the studies of Manning, McIntyre, Lutchmaya and colleagues [3], [4], [6], [7], [47], [57]–[59], only EPA female monkeys exhibit a significant difference in finger length ratio. In contrast to an expected smaller 2D∶4D finger length ratio, however, EPA females demonstrate a relatively larger 2D∶4D ratio finger length ratio compared to both normal control females and males, yet only in their right hands. This unexpected hyper-feminine ∼8% increase in right hand 2D∶4D ratio in EPA female monkeys is likely the product of an ∼7% elongation in 2D finger length. The degree of increased finger length in EPA females is comparable to the ∼11% greater length of 2D found in the right hand of normal males compared to control females. Right hand 2D elongation in EPA females, however, is without the accompanying ∼13% longer right hand 4D, as found in males, hence the increased 2D∶4D ratio in EPA females, but not in males.

EPA 2D finger elongation is highly predictive of the degree of masculinized (elongated) anogenital distance in EPA females. Such a predictive relationship between the degree of masculinized genitalia and right hand 2D finger length heavily contributes to the positive association between right hand 2D∶4D finger length ratio and anogenital distance. The correlation with degree of masculinized genitalia remains for right hand 2D finger length, but not 2D∶4D, when males are included. Female right hand 2D finger length also positively correlates with duration of fetal T exposure, when LPA females are excluded (LPA anogenital distance is unresponsive to T). 2D finger elongation in EPA females thus likely reflects the action of fetal T, or its locally derived androgenic or estrogenic metabolites, on finger traits determined during the critical period of early-to-mid gestation. Anogenital distance is crucial in providing understanding for this potential fetal mechanism since in rhesus monkeys, as in humans, anogenital distance is an established biomarker of the duration of fetal T exposure (as confirmed by this study), mostly through the action of its locally derived androgenic metabolite, dihydrotestosterone, during early-to-mid, but not late, gestation [29], [30], [32], [33], [60]. EPA female rhesus monkeys display male-like elongation of their anogenital distance in addition to obvious virilization of their external genitalia (phallus and scrotum). Thus, in female rhesus monkeys during early-to-mid gestation, duration of exposure to fetal male-typical T levels incrementally increases both anogenital distance and right hand 2D finger length, suggesting that androgen action on the two anatomical differentiation events may be temporally linked.

### Right-sided bias for 2D∶4D

Right-sided bias in digit ratio differences also occurs in humans, other nonhuman primates, and non-primate mammals [24], [25], [61], [62]. In humans, the magnitude of the sex difference in 2D∶4D can be greater in the right than the left hand [18], [51], [62], possibly because male-typical fetal T levels increase the degree of bias in right-sided lateralization together with lower 2D∶4D [18], [51], [63]–[65]. Right-sided bias in human 2D∶4D is positively associated with tissue oxygen uptake and physical performance [62], [66], [67] and may therefore contribute survival advantages. Studies, however, are inconsistent as to whether right-sided bias in human 2D∶4D is [62], [68]–[72] or is not [73], [74] associated with an increase in left-handedness as part of a functional asymmetry and cerebral lateralization [75]. A larger right hand 2D∶4D, however, is associated with right handedness in both men and women [70]. Monkey handedness was not determined in the current study.

### Potential mechanisms of fetal T induced changes in 2D∶4D

Sexually dimorphic 2D∶4D finger length ratios are not unusual in nonhuman primates [58], [76] and manifest together with additional sexually dimorphic ratios for phalanges, metacarpals and metatarsals in both hands and feet [77], [78]. Sexual dimorphism in baboon, gorilla and chimpanzee metacarpal and metatarsal bone length and ratios suggest T-mediated effects on a variety of genes regulating phalanx growth [24], [77], [78]. In the present study, however, in which female rhesus monkeys exposed to fetal T during early-to-mid, but not late, gestation exhibit elongated right hand 2D finger length and increased right hand 2D∶4D ratio, T-exposed EPA females show no corresponding changes in phalanx length or joint space width, suggesting insufficient involvement of bone, cartilage and joint connective tissue in mediating elongation of right hand 2D finger length. As rhesus monkeys, typical of anthropoid primates, have obvious adipose accumulation in pronounced fingertip pads retained from fetal life [79], and EPA females exhibit differential accumulation of abdominal fat [38], [80] and masculinized skin whorls in fingertip pads [79], [81], [82], early-to-mid fetal T exposure may direct preferential accumulation of adipose to the right hand 2D fingertip in EPA females, potentially through a mechanism similar to that proposed for 2D∶4D sex differences in humans [83], [84]. Subtle, menstrual cycle dependent changes in 2D∶4D in women certainly suggest contributions of soft tissue to female finger length [85].

With regard to a T-dependent mechanism regulating finger length, Talarovicova and colleagues [86] have shown in rats that fetal T exposure diminishes 2D length in the left forepaw and elongates 4D length in both forepaws resulting in the expected smaller 2D∶4D ratios. In mice, Zheng and Cohn [25] elegantly demonstrate the relevance of both androgen receptor (AR) and estrogen receptor alpha (ERα) in regulating developing digit primordia, focusing on the hind paws. Through fetal exposure to DHT or elimination of ERα expression in limbs of female mice, Zheng and Cohn [25] show that androgen excess or estrogen absence elongates 4D length and reduces 2D∶4D ratio. For male mice, elimination of AR expression in limbs or fetal exposure to the androgen antagonist, flutamide, or estradiol, demonstrate that absence of androgen action or presence of estrogen excess diminishes 4D length and increases the 2D∶4D ratio, and provide converse hormonal and developmental findings to those in female mice. The mouse findings reinforce the importance of fetal effects of both androgenic and estrogenic action on finger length found previously in a human study associating a higher amnionic fluid ratio of testosterone to estradiol with reduced 2D∶4D finger length ratio [6]. The mouse studies also find that expression of both AR and ERα are greater in 4D compared to 2D in males and females [25]. Results of the current monkey study, however, fail to emulate both rat and mouse findings with regard to right hand 2D finger length differences in EPA females, but do emulate these previous rodent results with regard to hindpaws [4], [25], as male rhesus monkeys have a smaller right foot 2D∶4D, achieved by an elongated 4D toe length.

Male-female monkey sexual dimorphism found in the right foot 2D∶4D in the present study is surprising in two regards. Firstly, our macaque sexual dimorphism is in the opposite direction from previously recorded human toe sexual dimorphism [48], [87]. Secondly, neither EPA nor LPA female monkeys show differences in right foot 2D∶4D ratio or 2D toe length compared to control females. Since fingers and toes develop at the same early-to-mid gestational age [88], the smaller 2D∶4D toe length ratio in only the male monkey right foot contrasts unexpectedly with an elongated 2D length and larger 2D∶4D ratio in only the EPA female monkey right hand, suggesting sexually dimorphic digit responses to fetal T exposure. In addition, the direction of sex differences in digit responses to fetal androgen or estrogen exposure, demonstrated in the earlier mouse study [25], is only emulated by male monkeys in the current study. One resolution of these digit differences between male and female monkeys could be achieved if a temporally discrete effect of fetal T (and/or its estrogenic metabolites) during mid-gestation (days 76–93 of gestation), a period of female monkey fetal development not examined by this study, was crucial for T-mediated elongation of 4D in both right hand and foot. Such gestational temporal differences in sex hormone-regulated 2D and 4D growth are possible since finger lengths reach their term length late in gestation [53].

There are, however, several additional potential explanations. Sexually dimorphic expression of AR and ERα may contribute to sex differences in the length of 4D [25]. Relatively more ERα expression in females [25] and local aromatization of exogenous T shortens 4D and relatively more AR in males [25] enables testicular fetal T to stimulate a longer 4D. In such a scenario, increased TP-injected monkey dam conjugation of estrogens due to placental aromatization of exogenous T in EPA pregnancies [56], combined with placental transport of sulfated estrogens and high expression of sulfatase in fetal digits [89], may increase 4D exposure to local estrogenic action and thus diminish T-mediated 4D elongation in EPA females, alone. Genetically-determined effects, independent of androgenic (or estrogenic) action, possibly involving interactions between testis-determining *SRY* and genes regulating finger length, such as *Sox9* the downstream target of *SRY* [25], and the interaction of *Sox9* with TGF-beta regulation of fetal digit extra-cellular matrix [90], may be crucial for male-like digit responses to the fetal steroid hormone environment. Regarding TGF-beta signaling in fetal digit development [90], the epigenetic profile of visceral adipose from both infant and adult EPA female monkeys includes altered DNA methylation of genes involving TGF-beta signaling [41]. Whether one or more of these potential mechanisms operate through combined effects on phalanx length, joint space width and fingertip adipose deposition remains to be determined.

The current pilot monkey study, however, does not differentiate between these and other possibilities, but does identify difficulty in employing adult 2D∶4D digit ratios as reliable, generic biomarkers for fetal T excess. Origins of digit length ratios are not as straightforward as initially proposed by Manning and colleagues [3], [4], [61], [91]. Our monkey results may help to explain why associations of 2D∶4D with developmental outcomes in humans are more pronounced than the magnitude of sex differences in 2D∶4D [62], [92], as well as the substantial variations in 2D∶4D between different human populations [58], [62]. As genetically-determined sex of an individual [93], together with gestational stage at fetal T exposure (from exogenous or endogenous sources) and its duration, may all influence how 2D∶4D manifests after birth, our monkey results call into question the widespread use of 2D∶4D as an associated fetal T biomarker implicating gestational T exposure with pathological [3], [10], [11], [18], [19] or other outcomes [3], [59], [61], [62]. Until *in utero*, hormonally-regulated mechanisms determining finger lengths in both sexes are elucidated in primates, including humans, our results indicate the potentially misleading nature of using adult 2D∶4D, alone, as a biomarker for fetal T exposure.

### Relevance of 2D∶4D to PCOS

Our pilot monkey study also permits re-interpretation of recent 2D∶4D findings in PCOS women and one previous conclusion that PCOS is not associated with prior gestational T exposure [94], [95]. In female animal models, while gestational T exposure reliably induces PCOS-like traits [34], [36], [96]–[98], the origins of PCOS in women are still unknown [35]. PCOS is a prevalent hyperandrogenic disorder of reproduction and metabolism in reproductive aged women [99]–[101]. Due to the difficulty of measuring human fetal T levels during gestation [19], [102], most cited evidence for fetal T exposure, such as 2D∶4D, is indirect. The use of adult 2D∶4D, however, as evidence for T exposure during gestation in women with PCOS has generated mixed results. Cattrall and colleagues [17] measured 2D∶4D in a group of 17 women with classic PCOS (selected by NIH criteria [103]) and discovered a small, but significant, decrease towards a male-like ratio in both left and right hands. Lujan and colleagues [94], [95], however, showed that women with a variety of PCOS phenotypes (Rotterdam criteria [46]) do not demonstrate a more male-like 2D∶4D in either left or right hands in any PCOS phenotype. Interestingly, however, PCOS women in Lujan's studies [94], [95] do exhibit a hyper-feminized 2D∶4D ratio because of relatively lengthy 2D compared to 4D finger lengths in both left and right hands. The greatest relative elongation of 2D finger length occurs in the most hyperandrogenic PCOS women, resulting in positive rather than the expected negative correlations between 2D∶4D and basal T, free androgen index and hirsutism score. 2D∶4D, however, is regulated mostly by the fetal and not adult hormone environment [7], [49]. Thus, if 2D∶4D is determined similarly in PCOS women as in EPA, PCOS-like female rhesus monkeys, such positive correlations between adult 2D∶4D and parameters of adult hyperandrogenism suggest that the degree of androgen excess and elongation of 2D∶4D in PCOS may reflect the degree and duration of fetal T exposure during early-to-mid gestation.

Direct evidence for a fetal T contribution to developmental origins of PCOS in humans, however, has been restricted to assessment of umbilical cord blood hormone levels from term births. Daughters born to women with PCOS, and at increased risk of PCOS in adulthood [104], have elevated, male-like T levels in umbilical vein blood at term [105]. In a separate study, however, PCOS daughters had reduced levels of androstenedione, an androgenic precursor to T, in mixed cord blood [106]. The late term gestational environment of PCOS daughters may thus be abnormal in terms of circulating androgens, but inconsistently so. Mixed cord blood levels of T are also not elevated in girls who were subsequently diagnosed with PCOS in adolescence [107]; however, the overly-prevalent adolescent diagnosis of PCOS (28%) in this Australian population is confounded by age-appropriate anovulation and multifollicular ovaries [108], [109]. Such term assessments, however, may be too removed from transient, mid-gestational ovarian androgen biosynthesis [110] and fetal male-like elevations in circulating T [102] to accurately identify PCOS risk. Accompanying elevations in maternal [111] or fetal [112] insulin during hyperglycemic gestations in PCOS women [113] and PA monkeys [112] may enhance fetal ovarian androgenicity [114]. Until advances in technology permit safe and accurate measurement of human fetal blood concentrations, or identification of a reliable postnatal biomarker of early-to-mid-gestational androgen exposure, understanding fetal T contributions to human 2D∶4D and to the origins of PCOS will remain elusive.

## Materials and Methods

### Ethics Statement

The Institutional Animal Care and Use Committee of the Graduate School of the University of Wisconsin-Madison approved all procedures used in the study, and the care and housing of the monkeys was in accordance with the recommendations of the Guide for the Care and Use of Laboratory Animals and Animal Welfare Act with its subsequent amendments.

### Animals

The 33 female and 9 male adult rhesus monkeys (*Macaca mulatta*) used in this study were maintained at WNPRC, according to standard protocol as previously described [30], [40]. Age, weight and body mass index (BMI; body weight (kg)/crown-rump length (m^2^) [115]) of female groups were comparable, whereas male body weight and BMI showed species-typical sexual dimorphism (Table 1). Somatometric measurements were obtained from each animal while anesthetized with ketamine HCl (7 mg/kg, intra-muscular (i.m.) injection) and xylazine (Rompun; 0.6 mg/kg, i.m).

Fourteen of the 33 female monkeys were exposed to fetal testosterone excess by subcutaneous (s.c.) injection of their dams with 10 mg testosterone propionate (TP). Dams of seven PA females received daily TP injections starting on gestational days 40–44 for 15–35 consecutive days (early-to-mid gestation, E). The other seven dams received injections of TP starting on gestational days 94–115 for 15–25 consecutive days (late gestation, L). We could only study 14 of the 23 previously described PA monkeys [37] as nine of the PA females had died of natural causes. The other 19 control female monkeys, and all males, in this study were not exposed to exogenous testosterone excess *in utero*, and were selected from monkeys not otherwise manipulated during gestation by other investigators or colony management at the WNPRC.

In some of the female monkeys employed in this study, somatometric measures, basal testosterone, menstrual cycle duration and ovarian morphology were previously reported in a variety of earlier studies, but are included here to provide appropriate context for analyses of the lengths of digits (fingers and toes) as well as phalanges (bones of the fingers and toes) and joint space width [34]–[37], [40], [116]–[118]. Blood samples providing serum for hormone analyses were obtained from animals trained to use a tabletop restraint without anesthesia [116]. Ovarian morphology was visualized during abdominal laparoscopy [119] while the animals were sedated with Ketamine HCl (10 mg/kg, i.m.).

### Parameters relevant to PCOS-like traits

#### Menstrual cycle assessment

Each female monkey underwent saphenous venipuncture three times weekly between 06:00 and 09:00 h while in a familiar tabletop restraint without anesthesia, and the resultant serum was assayed for progesterone for ∼2–6 month intervals [34]–[37], [40], [116]–[118]. Since menstrual discharge was not usually observed in approximately one-third of ovulatory EPA female rhesus monkeys [116], both a decline in serum progesterone values and the first day of menstruation were used to determine menstrual cycle phase onset and duration. The day that serum progesterone levels exceeded 0.4 ng/ml was designated as the first day of a luteal phase, while the day that serum progesterone levels declined below 0.4 ng/ml was defined as the onset of the follicular phase [116]. Ovulatory menstrual cycles were identified as those with two serum progesterone levels above 1 ng/ml within 15 days before menses or serum progesterone falling below 0.4 ng/ml [30], [116].

#### Hormone assays

Circulating progesterone and T determinations were undertaken by enzymeimmunoassay in the WNPRC/Institute of Clinical Translational Research (ICTR) Hormone Assay Services Laboratory [117], [120]. T measurements were performed following diethyl ether extraction of serum and solvent fraction separation by celite chromatography. Intra- and inter-assay CVs for quality control preparation (QC) values were, respectively, progesterone, 3.9% and 8.9%; T, 3.5% and 14.0%.

#### Ovarian morphology

During laparoscopic assessment of ovarian dimensions [121], [122] while the animals were sedated with Ketamine HCl (10 mg/kg, i.m.), photographic images were taken of trans-illuminated ovaries [119] at their largest diameter during the early follicular phase (menstrual cycle days 1–5) or an anovulatory interval. Ovarian images with >10, ∼1–3 mm diameter follicles were scored as polyfollicular (Table 1), a criterion modified from the prevailing ultrasonographic determination of polycystic ovaries in women [45] before the Rotterdam consensus [46].

### Somatometrics

Somatometric measurements were performed immediately after x-rays of hands and feet, or after DXA scans (for an unrelated study), as previously validated for rhesus monkeys [123]. Each animal was anesthetized with ketamine HCl (7 mg/kg, i.m.) and xylazine (Rompun; 0.6 mg/kg, i.m) and was assessed for body weight, crown-rump length, digit length and anogenital distance.

#### Digit Lengths

With the animal in left lateral recumbency, digit measurements were taken of the right hand and foot. The animal was then moved to right lateral recumbency and the left hand and foot digits were measured. Digit lengths were measured on the ventral surface of the hands and feet, using a Lange digitized caliper measuring to the nearest ±0.1 mm, from the middle of the proximal skin crease at the base of the digit to the mid-point at the tip of the digit [3], [4], [58]. Each digit was extended and placed flat on a tabletop, dorsal side facing down, during measurement to ensure the most accurate digit measurement possible, while avoiding the confounding factor of soft tissue in the finger tips distorting the length measurement when pressed ventrally against a glass surface to be photocopied or scanned [51]. The same experimenter repeated the digit measurement three times for all monkeys, taking the average for each digit. Because of age and social housing, several digit measures were omitted due to either visibly compromised digits (i.e., incomplete, damaged, missing, bent) or x-ray determined damage in digit phalanges (i.e., arthritic growth, dislocated bones, improperly healed fractures, missing bones, broken bones). Interclass correlation coefficient (ICC) was used to assess reliability of mean finger and toe length measurement (average length ICC with absolute-agreement definition) [124] and ranged from 0.95–0.98.

#### Anogenital distance

This measure was performed with the animal in right lateral recumbency and using a cloth tape measure to the nearest ±0.1 cm. One end of the tape measure was placed above the center of the anus while the length measurement above the center of the urethra was recorded.

#### Phalanx measurements

Radiographs, using standard techniques, were taken of hands and feet of all monkeys with digits fully extended and flattened against the radiographic plate. Three radiographs of the right foot in control females were omitted from analyses because they did not permit accurate phalanx measurements when viewed under ×2 magnification on a radiograph light box. A single operator measured each phalanx (proximal, P1; intermediate, P2; distal, P3) three different times to obtain the average length used in analyses while blind to female fetal history. Phalanx length was obtained using a Fisherbrand Traceable Electronic Digital Caliper accurate to ±0.01 mm from the mid-points of the proximal and distal ends of each phalanx [2]. Emphasis was placed on measuring the straight alignment of the distal and proximal ends of the shaft rather than its vertical alignment [78]. The same observer, after ∼2–60 months, used the same methodology to re-measure phalanges in 76.2% (32/42) of radiographs, without regard to previous measurements, in order to assess reliability of phalanx measurements by calculating intra-observer reliability (IOR) between original and repeated assessments [57], [58]. Intra-observer correlations ranged from 0.95–0.97.

Due to the mid-to-late reproductive years of the monkeys used [36], [37], an independent observer scored all the phalanges for arthritis while blind to animal group. Digits that were obviously arthritic, damaged, missing, broken, incomplete or bent were omitted from analyses.

#### Cartilage Measurements

The joint space width (JSW) of the metacarpophalangeal (MCP), proximal interphalangeal (PIP) and distal interphalangeal (DIP) joints in the second digit of the right hand were measured from the already captured radiographs using a Fisherbrand Traceable Electronic Digital Caliper, accurate to ±0.01 mm. Using the previously described method of measuring JSW by Angwin and colleagues [125], values were taken from three different positions along the JSW: two were on the outside of the second digit – lateral and medial to the middle finger, and one in the center of the JSW of the second digit [125]. A single operator measured each JSW three different times to obtain the average length used in analyses while blind to animal group. The same observer, after 6 months, used the same methodology to re-measure JSW in 31% (13/42) of radiographs, without regard to previous measurements. Intra-observer correlations ranged from 0.65–0.71.

### Statistical Analysis

Variables were compared by one-way ANOVA using fetal T exposure as the main factor. When significant (p<0.05), post-hoc analysis was performed using Tukey's test (Systat 12, Chicago, IL). Least-mean square regression was employed to examine parameter association. As a large number of ANOVAs were performed on phalanx and joint distance measures, the standard criterion for statistical significance (p<0.05) may have been exceeded by chance. It is thus important that statistical assessments of phalanx and joint space parameters be interpreted in relation to appropriate accompanying effect size (*η* ^2^_p_; [43], [44]), particularly medium (∼0.5) to large (∼0.8) effect sizes [42], as employed by McFadden and Bracht [126] in examining relative lengths of metacarpals and metatarsals in Great Apes. Effect sizes for all parameters are provided in Table S3.

## Supporting Information

Table S1 **Mean (± SEM) phalanx lengths in control, early (EPA) and late (LPA) prenatally androgenized female and control male rhesus monkeys.**
(DOCX)

Table S2 **Mean (± SEM) phalanx length ratios in control, early (EPA) and late (LPA) prenatally androgenized female and control male rhesus monkeys.**
(DOCX)

Table S3 **Effect size (** ***η*** **^2^_p_)** [**42**] **of digit length averages, phalanx length averages, digit ratios, phalanx length ratios and biological statistics in control, early (EPA) and late (LPA) prenatally androgenized female and control male rhesus monkeys.** Categories of effect size: small: 0.20; medium: 0.50; large: 0.80 [42].
(DOCX)
