# Supplementary material for: Early-to-Mid Gestation Fetal Testosterone Increases Right Hand 2D∶4D Finger Length Ratio in Polycystic Ovary Syndrome-Like Monkeys
Source: PLoS One. 2012 Aug 22;7(8):e42372. doi: 10.1371/journal.pone.0042372 (PMC3425513; doi:10.1371/journal.pone.0042372)
Supplement: Table S1 — Mean (± SEM) phalanx lengths in control, early (EPA) and late (LPA) prenatally androgenized female and control male rhesus monkeys. (DOCX) [file pone.0042372.s001.docx]

**Table S1.** Mean (± SEM) phalanx lengths in control, early (EPA) and late (LPA) prenatally androgenized female and control male rhesus monkeys.

|  | **Control Female** | **EPA** | **LPA** | **Male** |
| --- | --- | --- | --- | --- |
| **Left Hand** |  |  |  |  |
| Phalanx 1: 2D | 1.986 ± 0.023^c^ | 2.005 ± 0.038^e^ | 1.978 ± 0.035^i^ | 2.199 ± 0.031 |
| Phalanx 2: 2D | 1.089 ± 0.020^b^ | 1.149 ± 0.035 | 1.107 ± 0.029^g^ | 1.222 ± 0.027 |
| Phalanx 3: 2D | 0.601 ± 0.014^c^ | 0.610 ± 0.023^e^ | 0.631 ± 0.022^h^ | 0.738 ± 0.020 |
| Phalanx 1-3: 2D | 3.678 ± 0.049^c^ | 3.758 ± 0.082^e^ | 3.717 ± 0.070^i^ | 4.158 ± 0.065 |
| Phalanx 1: 3D | 2.398 ± 0.023^c^ | 2.387 ± 0.037^f^ | 2.367 ± 0.037^i^ | 2.652 ± 0.033 |
| Phalanx 2: 3D | 1.444 ± 0.036^a^ | 1.454 ± 0.053 | 1.439 ± 0.048^g^ | 1.621 ± 0.042 |
| Phalanx 3: 3D | 0.735 ± 0.022^b^ | 0.710 ± 0.042^e^ | 0.720 ± 0.037^h^ | 0.894 ± 0.030 |
| Phalanx 1-3: 3D | 4.552 ± 0.065^c^ | 4.658 ± 0.108^e^ | 4.606 ± 0.096^h^ | 5.149 ± 0.076 |
| Phalanx 1: 4D | 2.353 ± 0.025^c^ | 2.339 ± 0.039^f^ | 2.328 ± 0.042^i^ | 2.607 ± 0.034 |
| Phalanx 2: 4D | 1.389 ± 0.026^c^ | 1.468 ± 0.050 | 1.450 ± 0.042 | 1.605 ± 0.039 |
| Phalanx 3: 4D | 0.793 ± 0.018^c^ | 0.780 ± 0.031^e^ | 0.791 ± 0.026^h^ | 0.942 ± 0.025 |
| Phalanx 1-3: 4D | 4.512 ± 0.065^c^ | 4.614 ± 0.105^e^ | 4.570 ± 0.096^i^ | 5.159 ± 0.083 |
|  |  |  |  |  |
| **Right hand** |  |  |  |  |
| Phalanx 1: 2D | 1.999 ± 0.022^c^ | 2.019 ± 0.033^e^ | 1.989 ± 0.033^i^ | 2.180 ± 0.029 |
| Phalanx 2: 2D | 1.081 ± 0.024^a^ | 1.116 ± 0.035 | 1.112 ± 0.038 | 1.206 ± 0.031 |
| Phalanx 3: 2D | 0.614 ± 0.015^c^ | 0.640 ± 0.024^e^ | 0.657 ± 0.024^h^ | 0.766 ± 0.019 |
| Phalanx 1-3: 2D | 3.695 ± 0.056^c^ | 3.803 ± 0.082^d^ | 3.747 ± 0.082^h^ | 4.150 ± 0.067 |
| Phalanx 1: 3D | 2.401 ± 0.033^c^ | 2.410 ± 0.051^d^ | 2.380 ± 0.051^h^ | 2.632 ± 0.042 |
| Phalanx 2: 3D | 1.436 ± 0.033 | 1.466 ± 0.046 | -^*^ | 1.608 ± 0.034 |
| Phalanx 3: 3D | 0.693 ± 0.029^b^ | 0.772 ± 0.045 | 0.797 ± 0.058 | 0.868 ± 0.033 |
| Phalanx 1-3: 3D | 4.543 ± 0.088 | 4.672 ± 0.118 | -^*^ | 5.100 ± 0.088 |
| Phalanx 1: 4D | 2.349 ± 0.027^c^ | 2.328 ± 0.043^f^ | 2.337 ± 0.040^i^ | 2.590 ± 0.035 |
| Phalanx 2: 4D | 1.371 ± 0.035^c^ | 1.454 ± 0.042 | 1.427 ± 0.047^g^ | 1.598 ± 0.035 |
| Phalanx 3: 4D | 0.759 ± 0.032^b^ | 0.772 ± 0.045 | 0.798 ± 0.045 | 0.927 ± 0.034 |
| Phalanx 1-3: 4D | 4.414 ± 0.096^c^ | 4.528 ± 0.113^e^ | 4.554 ± 0.113^h^ | 5.116 ± 0.084 |
|  |  |  |  |  |
| **Left foot** |  |  |  |  |
| Phalanx 1: 2D | 2.127 ± 0.029 | 2.162 ± 0.044 | 2.247 ± 0.063 | 2.238 ± 0.038 |
| Phalanx 2: 2D | 1.104 ± 0.030 | 1.106 ± 0.044 | 1.090 ± 0.053 | 1.194 ± 0.044 |
| Phalanx 3: 2D | 0.717 ± 0.015^c^ | 0.687 ± 0.024^f^ | 0.763 ± 0.026^h^ | 0.885 ± 0.023 |
| Phalanx 1-3: 2D | 3.935 ± 0.059^b^ | 3.993 ± 0.086^d^ | 4.217 ± 0.012 | 4.326 ± 0.080 |
| Phalanx 1: 3D | 2.568 ± 0.031^b^ | 2.586 ± 0.048^d^ | 2.558 ± 0.052^g^ | 2.768 ± 0.043 |
| Phalanx 2: 3D | 1.522 ± 0.026 | 1.588 ± 0.044 | 1.506 ± 0.040 | 1.640 ± 0.035 |
| Phalanx 3: 3D | 0.853 ± 0.025^b^ | 0.892 ± 0.042 | 0.878 ± 0.042 | 1.028 ± 0.037 |
| Phalanx 1-3: 3D | 4.915 ± 0.070^c^ | 5.100 ± 0.122 | 4.953 ± 0.111^g^ | 5.438 ± 0.096 |
| Phalanx 1: 4D | 2.452 ± 0.027^c^ | 2.470 ± 0.042^d^ | 2.463 ± 0.042^h^ | 2.651 ± 0.037 |
| Phalanx 2: 4D | 1.483 ± 0.022^b^ | 1.524 ± 0.032 | 1.509 ± 0.034 | 1.617 ± 0.03 |
| Phalanx 3: 4D | 0.860 ± 0.028^c^ | 0.884 ± 0.043^e^ | 0.873 ± 0.046^h^ | 1.082 ± 0.040 |
| Phalanx 1-3: 4D | 4.781 ± 0.070^c^ | 4.883 ± 0.099 | 4.867 ± 0.107^h^ | 5.358 ± 0.093 |
|  |  |  |  |  |
| **Right foot** |  |  |  |  |
| Phalanx 1: 2D | 2.130 ± 0.030 | 2.086 ± 0.050^d^ | 2.137 ± 0.054 | 2.266 ± 0.044 |
| Phalanx 2: 2D | 1.126 ± 0.027 | 1.084 ± 0.052 | 1.087 ± 0.047 | 1.183 ± 0.039 |
| Phalanx 3: 2D | 0.726 ± 0.016^c^ | 0.695 ± 0.029^f^ | 0.721 ± 0.027^h^ | 0.856 ± 0.024 |
| Phalanx 1-3: 2D | 3.982 ± 0.063^a^ | 3.842 ± 0.120^d^ | 3.937 ± 0.110 | 4.301 ± 0.090 |
| Phalanx 1: 3D | 2.586 ± 0.030^a^ | 2.568 ± 0.057 | 2.561 ± 0.048^g^ | 2.750 ± 0.042 |
| Phalanx 2: 3D | 1.514 ± 0.020^b^ | 1.523 ± 0.042 | 1.499 ± 0.034^g^ | 1.637 ± 0.030 |
| Phalanx 3: 3D | 0.889 ± 0.026 | 0.874 ± 0.049 | 0.872 ± 0.045 | 0.986 ± 0.041 |
| Phalanx 1-3: 3D | 4.961 ± 0.069^a^ | 4.958 ± 0.138 | 4.974 ± 0.123 | 5.354 ± 0.104 |
| Phalanx 1: 4D | 2.478 ± 0.027^b^ | 2.452 ± 0.053^d^ | 2.441 ± 0.045^h^ | 2.662 ± 0.039 |
| Phalanx 2: 4D | 1.486 ± 0.022^a^ | 1.455 ± 0.048 | 1.510 ± 0.039 | 1.605 ± 0.034 |
| Phalanx 3: 4D | 0.891 ± 0.021^c^ | 0.900 ± 0.045^d^ | 0.873 ± 0.037^h^ | 1.058 ± 0.032 |
| Phalanx 1-3:4D | 4.847 ± 0.064^b^ | 4.798 ± 0.137^d^ | 4.822 ± 0.112^h^ | 5.345 ± 0.097 |

Phalanx 1-3: Combined lengths of phalanges 1-3.

* There were only two LPA females with an intact phalanx 2 in the 3^rd^ digit of their right hand.

^a^ Control Female < Male, 0.01 < p < 0.05 ^f^ EPA < Male, p < 0.001

^b^ Control Female < Male, 0.001 < p < 0.01 ^g^ LPA < Male, 0.01 < p < 0.05

^c^ Control Female < Male, p < 0.001 ^h^ LPA < Male, 0.001 < p < 0.01

^d^ EPA < Male, 0.01 < p <0.05 ^i^ LPA < Male, p < 0.001

^e^ EPA < Male, 0.001 < p < 0.01
