# Supplementary material for: Early-to-Mid Gestation Fetal Testosterone Increases Right Hand 2D∶4D Finger Length Ratio in Polycystic Ovary Syndrome-Like Monkeys
Source: PLoS One. 2012 Aug 22;7(8):e42372. doi: 10.1371/journal.pone.0042372 (PMC3425513; doi:10.1371/journal.pone.0042372)
Supplement: Table S2 — Mean (± SEM) phalanx length ratios in control, early (EPA) and late (LPA) prenatally androgenized female and control male rhesus monkeys. (DOCX) [file pone.0042372.s002.docx]

**Table S2.** Mean (± SEM) phalanx length ratios in control, early (EPA) and late (LPA) prenatally androgenized female and control male rhesus monkeys.

|  | **Control Female** | **EPA** | **LPA** | **Male** |
| --- | --- | --- | --- | --- |
| **Left Hand** |  |  |  |  |
| Phalanx 1 2D:3D | 0.827 ± 0.004 | 0.845 ± 0.007 | 0.834 ± 0.007 | 0.829 ± 0.006 |
| Phalanx 2 2D:3D | 0.755 ± 0.011 | 0.802 ± 0.017 | 0.773 ± 0.014 | 0.755 ± 0.012 |
| Phalanx 3 2D:3D | 0.812 ± 0.034 | 0.880 ± 0.062 | 0.896 ± 0.056 | 0.832 ± 0.044 |
| Phalanx 1-3 2D:3D | 0.800 ± 0.008 | 0.830 ± 0.014 | 0.814 ± 0.011 | 0.805 ± 0.008 |
| Phalanx 1 2D:4D | 0.853 ± 0.005 | 0.863 ± 0.008 | 0.860 ± 0.008 | 0.844 ± 0.006 |
| Phalanx 2 2D:4D | 0.779 ± 0.010 | 0.793 ± 0.019 | 0.766 ± 0.014 | 0.762 ± 0.014 |
| Phalanx 3 2D:4D | 0.763 ± 0.022 | 0.798 ± 0.041 | 0.807 ± 0.031 | 0.786 ± 0.029 |
| Phalanx 1-3 2D:4D | 0.812 ± 0.007 | 0.830 ± 0.010 | 0.820 ± 0.009 | 0.808 ± 0.007 |
| Phalanx 1 3D:4D | 1.022 ± 0.064 | 1.023 ± 0.009 | 1.025 ± 0.010 | 1.019 ± 0.009 |
| Phalanx 2 3D:4D | 1.038 ± 0.019 | 0.998 ± 0.030 | 0.988 ± 0.024 | 1.012 ± 0.021 |
| Phalanx 3 3D:4D | 0.944 ± 0.028 | 0.890 ± 0.057 | 0.860 ± 0.044 | 0.965 ± 0.035 |
| Phalanx 1-3 3D:4D | 1.014 ± 0.008 | 1.000 ± 0.013 | 0.995 ± 0.012 | 1.005 ± 0.008 |
|  |  |  |  |  |
| **Right hand** |  |  |  |  |
| Phalanx 1 2D:3D | 0.825 ± 0.007 | 0.843 ± 0.009 | 0.837 ± 0.009 | 0.829 ± 0.008 |
| Phalanx 2 2D:3D | 0.741 ± 0.012 | 0.782 ± 0.017 | -^*^ | 0.750 ± 0.012 |
| Phalanx 3 2D:3D | 0.902 ± 0.038 | 0.842 ± 0.056 | 0.810 ± 0.073 | 0.896 ± 0.042 |
| Phalanx 1-3 2D:3D | 0.813 ± 0.009 | 0.820 ± 0.011 | -^*^ | 0.814 ± 0.008 |
| Phalanx 1 2D:4D | 0.849 ± 0.006 | 0.873 ± 0.008 | 0.851 ± 0.008 | 0.843 ± 0.007 |
| Phalanx 2 2D:4D | 0.764 ± 0.016 | 0.778 ± 0.017 | 0.755 ± 0.021 | 0.754 ± 0.014 |
| Phalanx 3 2D:4D | 0.810 ± 0.026 | 0.842 ± 0.034 | 0.800 ± 0.038 | 0.831 ± 0.026 |
| Phalanx 1-3 2D:4D | 0.814 ± 0.010 | 0.832 ± 0.010 | 0.808 ± 0.011 | 0.811 ± 0.007 |
| Phalanx 1 3D:4D | 1.019 ± 0.008 | 1.033 ± 0.011 | 1.013 ± 0.011 | 1.016 ± 0.009 |
| Phalanx 2 3D:4D | 1.050 ± 0.014 | 0.998 ± 0.014 | -^*^ | 1.008 ± 0.011 |
| Phalanx 3 3D:4D | 0.914 ± 0.039 | 0.945 ± 0.052 | 0.980 ± 0.060 | 0.962 ± 0.034 |
| Phalanx 1-3 3D:4D | 1.000 ± 0.013 | 1.010 ± 0.012 | -^*^ | 1.001 ± 0.008 |
|  |  |  |  |  |
| **Left foot** |  |  |  |  |
| Phalanx 1 2D:3D | 0.841 ± 0.006 | 0.830 ± 0.010 | 0.855 ± 0.016 | 0.816 ± 0.008 |
| Phalanx 2 2D:3D | 0.735 ± 0.014 | 0.740 ± 0.021 | 0.740 ± 0.024 | 0.730 ± 0.018 |
| Phalanx 3 2D:3D | 0.837 ± 0.022 | 0.780 ± 0.037 | 0.900 ± 0.040 | 0.862 ± 0.032 |
| Phalanx 1-3 2D:3D | 0.807 ± 0.006 | 0.796 ± 0.009 | -^*^ | 0.798 ± 0.008 |
| Phalanx 1 2D:4D | 0.872 ± 0.008 | 0.866 ± 0.013 | 0.880 ± 0.017 | 0.848 ± 0.010 |
| Phalanx 2 2D:4D | 0.738 ± 0.016 | 0.724 ± 0.021 | 0.725 ± 0.028 | 0.744 ± 0.021 |
| Phalanx 3 2D:4D | 0.857 ± 0.031 | 0.781 ± 0.046 | 0.882 ± 0.055 | 0.821 ± 0.043 |
| Phalanx 1-3 2D:4D | 0.827 ± 0.008 | 0.813 ± 0.010 | -^*^ | 0.808 ± 0.010 |
| Phalanx 1 3D:4D | 1.043 ± 0.006 | 1.047 ± 0.009 | 1.033 ± 0.009 | 1.041 ± 0.008 |
| Phalanx 2 3D:4D | 1.007 ± 0.010 | 1.016 ± 0.014 | 1.002 ± 0.013 | 1.014 ± 0.011 |
| Phalanx 3 3D:4D | 1.045 ± 0.040 | 1.003 ± 0.064 | 1.005 ± 0.064 | 0.955 ± 0.055 |
| Phalanx 1-3 3D:4D | 1.024 ± 0.008 | 1.026 ± 0.011 | 1.018 ± 0.010 | 1.018 ± 0.009 |
|  |  |  |  |  |
| **Right foot** |  |  |  |  |
| Phalanx 1 2D:3D | 0.825 ± 0.006 | 0.832 ± 0.011 | 0.837 ± 0.010 | 0.825 ± 0.009 |
| Phalanx 2 2D:3D | 0.736 ± 0.014 | 0.730 ± 0.028 | 0.736 ± 0.026 | 0.731 ± 0.020 |
| Phalanx 3 2D:3D | 0.819 ± 0.024 | 0.826 ± 0.045 | 0.858 ± 0.041 | 0.893 ± 0.038 |
| Phalanx 1-3 2D:3D | 0.795 ± 0.008 | 0.800 ± 0.016 | 0.804 ± 0.014 | 0.810 ± 0.012 |
| Phalanx 1 2D:4D | 0.861 ± 0.008 | 0.832 ± 0.016 | 0.873 ± 0.015 | 0.851 ± 0.012 |
| Phalanx 2 2D:4D | 0.759 ± 0.015 | 0.748 ± 0.033 | 0.717 ± 0.027 | 0.732 ± 0.023 |
| Phalanx 3 2D:4D | 0.812 ± 0.017 | 0.772 ± 0.035 | 0.825 ± 0.029 | 0.825 ± 0.025 |
| Phalanx 1-3 2D:4D | 0.824 ± 0.009 | 0.798 ± 0.018 | 0.817 ± 0.015 | 0.811 ± 0.013 |
| Phalanx 1 3D:4D | 1.042 ± 0.006 | 1.040 ± 0.012 | 1.049 ± 0.009 | 1.033 ± 0.008 |
| Phalanx 2 3D:4D | 1.027 ± 0.009 | 1.013 ± 0.023 | 1.004 ± 0.018 | 1.002 ± 0.014 |
| Phalanx 3 3D:4D | 0.998 ± 0.026 | 0.963 ± 0.065 | 1.012 ± 0.046 | 0.956 ± 0.042 |
| Phalanx 1-3 3D:4D | 1.028 ± 0.006 | 1.020 ± 0.013 | 1.024 ± 0.010 | 1.010 ± 0.009 |

Phalanx 1-3: Ratio calculated from the combined phalanges lengths of each digit.

* There were only two LPA females with intact phalanges permitting calculation of 2D:3D and 3D:4D in the right hand, as well as 2D:3D and 2D:4D in the left foot.
