# Supplementary material for: Early-to-Mid Gestation Fetal Testosterone Increases Right Hand 2D∶4D Finger Length Ratio in Polycystic Ovary Syndrome-Like Monkeys
Source: PLoS One. 2012 Aug 22;7(8):e42372. doi: 10.1371/journal.pone.0042372 (PMC3425513; doi:10.1371/journal.pone.0042372)
Supplement: Table S3 — Effect size ( η 2p) [42] of digit length averages, phalanx length averages, digit ratios, phalanx length ratios and biological statistics in control, early (EPA) and late (LPA) prenatally androgenized female and control male rhesus monkeys. Categories of effect size: small: 0.20; medium: 0.50; large: 0.80 [42]. (DOCX) [file pone.0042372.s003.docx]

**Table S3.** Effect size (*η*^2^_p_) [42] of digit length averages, phalanx length averages, digit ratios, phalanx length ratios and biological statistics in control, early (EPA) and late (LPA) prenatally androgenized female and control male rhesus monkeys. Categories of effect size: small: 0.20; medium: 0.50; large: 0.80 [42].

| **Length *η*^2^_p_** | **Digit** | **Phalanx 1** | **Phalanx 2** | **Phalanx 3** | **Phalanges 1-3** |
| --- | --- | --- | --- | --- | --- |
| LH 2D | 0.36 | 0.52 | 0.34 | 0.50 | 0.56 |
| LH 3D | 0.54 | 0.58 | 0.33 | 0.47 | 0.62 |
| LH 4D | 0.47 | 0.57 | 0.38 | 0.49 | 0.60 |
| RH 2D | 0.48 | 0.47 | 0.24 | 0.56 | 0.49 |
| RH 3D | 0.57 | 0.43 | 0.40 | 0.39 | 0.50 |
| RH 4D | 0.64 | 0.53 | 0.47 | 0.38 | 0.63 |
| LF 2D | 0.45 | 0.20 | 0.10 | 0.57 | 0.42 |
| LF 3D | 0.35 | 0.32 | 0.24 | 0.32 | 0.41 |
| LF 4D | 0.34 | 0.38 | 0.30 | 0.41 | 0.46 |
| RF 2D | 0.06 | 0.20 | 0.09 | 0.42 | 0.27 |
| RF 3D | 0.32 | 0.27 | 0.30 | 0.14 | 0.28 |
| RF 4D | 0.45 | 0.36 | 0.24 | 0.41 | 0.40 |
|  |  |  |  |  |  |
| **Ratio *η*^2^_p_** | **Digit** | **Phalanx 1** | **Phalanx 2** | **Phalanx 3** | **Phalanges 1-3** |
| LH 2D:3D | 0.18 | 0.13 | 0.22 | 0.07 | 0.15 |
| LH 2D:4D | 0.07 | 0.12 | 0.07 | 0.05 | 0.13 |
| LH 3D:4D | 0.30 | 0.01 | 0.11 | 0.15 | 0.10 |
| RH 2D:3D | 0.28 | 0.09 | 0.20 | 0.07 | 0.03 |
| RH 2D:4D | 0.57 | 0.21 | 0.06 | 0.04 | 0.16 |
| RH 3D:4D | 0.28 | 0.07 | 0.32 | 0.06 | 0.05 |
| LF 2D:3D | 0.16 | 0.24 | 0.01 | 0.14 | 0.19 |
| LF 2D:4D | 0.04 | 0.15 | 0.02 | 0.08 | 0.12 |
| LF 3D:4D | 0.06 | 0.04 | 0.03 | 0.05 | 0.02 |
| RF 2D:3D | 0.33 | 0.04 | 0.01 | 0.08 | 0.04 |
| RF 2D:4D | 0.30 | 0.11 | 0.06 | 0.05 | 0.06 |
| RF 3D:4D | 0.22 | 0.04 | 0.08 | 0.04 | 0.10 |
|  |  |  |  |  |  |
| **Biological statistics** | ***η*^2^_p_** |  |  |  |  |
| Age | 0.29 |  |  |  |  |
| Body Weight | 0.53 |  |  |  |  |
| Crown-Rump | 0.35 |  |  |  |  |
| Body-Mass Index | 0.26 |  |  |  |  |
| Anogenital Distance | 0.95 |  |  |  |  |
| Female Basal Testosterone | 0.21 |  |  |  |  |
